# High genetic diversity and predominance of Rhinovirus A and C from Panamanian hospitalized children under five years with respiratory infections

**DOI:** 10.1186/1743-422X-9-257

**Published:** 2012-11-01

**Authors:** Danilo Franco, Adriana Delfraro, Leyda Abrego, Maria Cano, Celedonio Castillo, Marlene Castillo, Juan Castillo, Juan Pascale, Juan Arbiza

**Affiliations:** 1 Instituto Conmemorativo Gorgas de Estudios de la Salud, Panama City, Panamá; 2 Sección Virologia, Facultad de Ciencias, Universidad de la República, Iguá 4225, Montevideo 11400, Uruguay; 3 School of Medicine, University of Panama, Panama City, Panama

**Keywords:** Rhinovirus, Panama, Genetic diversity

## Abstract

**Background:**

Human Rhinoviruses (HRVs) have high genetic diversity and three species have been described: HRV-A, HRV-B, and the recently recognized HRV-C, which has been rapidly identified worldwide.

**Findings:**

In the present study, we report the frequency and diversity of Human Rhinovirus (HRV) strains circulating in Panama from children hospitalized with respiratory infections.

**Conclusions:**

HRVs of species A, B and C have been identified with a predominance of HRV-A and HRV-C over HRV-B, and marked genetic diversity within each species.

## Findings

Human rhinoviruses (HRVs) are the most common causative agents of upper respiratory tract infections, but are also associated with more severe diseases such as pneumonia or acute wheezing related to bronchiolitis and acute asthma in children [1-4]. HRV infection occurs in all age groups and is responsible for 25% to 50% of respiratory infections presenting as influenza-like illnesses [5-7].

HRVs have been classified into the genus *Enterovirus*, family *Picornaviridae*[8]. As other picornaviruses, they are small, non-enveloped viruses with a 7200 bp single-stranded, positive-sense mRNA genome and a long open reading frame encoding four capsid proteins VP4, VP2, VP3 and VP1, and seven non-structural proteins 2A, 2B, 2C, 3A, 3B, 3C and 3D.

HRVs have high genetic diversity and three species have been described: HRV-A, HRV-B, and the recently recognized HRV-C [9], which has been rapidly identified worldwide [10-12].

Reports that infection with different HRV species result in different clinical outcomes are controversial: some show correlations between a given viral serotype or species and its capacity to invade the upper or lower respiratory tract [13], whereas others provide evidence for a more frequent role for HRV-C in lower respiratory tract infections, associated with more severe disease [9,11]. In a recent study, HRV rates were high among hospitalized children and the elderly, but HRV was also detected among asymptomatic children. HRV-A and HRV-C were associated with illness requiring hospitalization [14].

Very little in general is known about the genetic diversity of respiratory viruses in Central America or Panama, nor specifically about rhinoviruses. In this study we investigate the genetic variability of Rhinovirus from children under five years, hospitalized in Panama with respiratory infections. Stored nasal swab samples were used in this study and the protocol was approved by Ethics Committee of Gorgas Institute. Sixty-two nasal swab samples positive for HRV by real time PCR [15] from a total 118 samples from 420 study subjects, collected from August 2010 (*n:* 178) to June 2011 (*n:* 242), were analyzed by RT-PCR and sequencing. Briefly, the viral RNA was extracted using QIAamp Viral RNA Mini Kit (Qiagen, GmbH, Hilden, Germany) according to the manufacturer’s instructions. Amplification of a 542 bp fragment containing the HRV VP4 and partial VP2 region was performed by RT-PCR using a reverse primer identical to that described previously [16] and external and semi-nested forward primers designed by Xiaoyan Lu and Dean Erdman at CDC (unpublished data). cDNA synthesis and PCR were carried out using QIAGEN OneStep RT-PCR Kit and Taq PCR Master Mix Kit and PCR products were purified using a Wizard® SV Gel and PCR Clean-Up System (Promega Corporation Madison, WI 53711 USA). Sequencing was performed in both directions using the same primers as in the PCR with an ABI Prism BigDye Terminator Cycle Sequencing Ready Reaction Kit according to the manufacturer’s instructions on a 3130XL Genetic Analyzer (Applied Biosystems, Foster City, CA).

Alignment and sequence analyses were performed using Bioedit, ClustalW and MEGA5 software. The most suitable model for nucleotide substitution was estimated with Modelgenerator. Phylogenetic trees were constructed under maximum likelihood criteria and branch supports were calculated by the approximate likelihood ratio test (aLRT). One hundred and fifty published HRV sequences within the VP4-VP2 region were obtained from the GenBank database (NCBI). The VP4/VP2 region was used to analyze the sequence variability within the much larger data set of published sequences of all HRV-A, HRV-B serotypes, and also from the recently described species C variants, which have been assigned to 28 provisional types [12].

**Figure 1:**
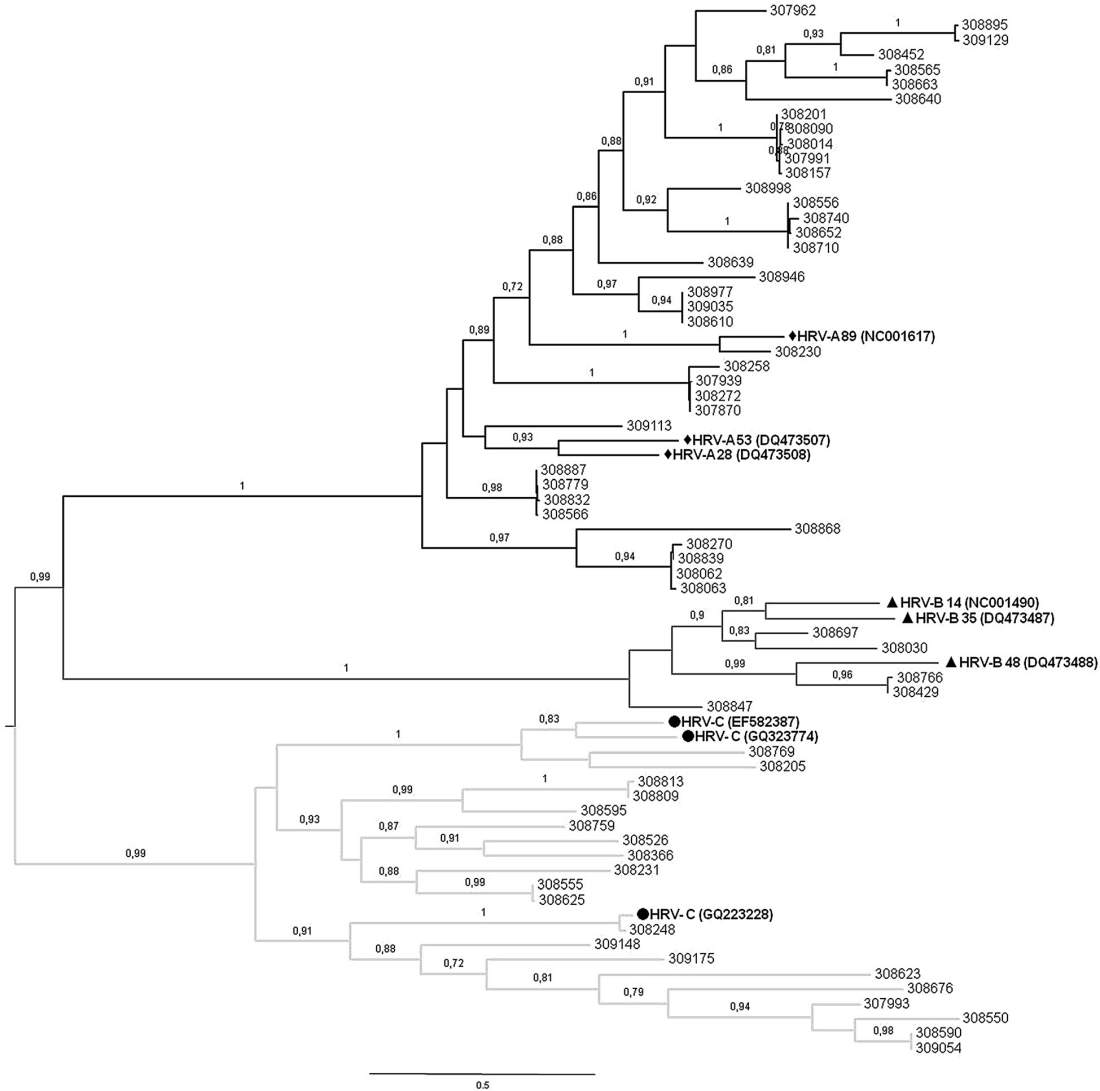
**Phylogenetic analysis of the VP4/VP2 gene from the 62 HRVs of this and reference strains for each species. **Phylogenetic trees were constructed by maximum likelihood by using PhyML. nucleotide substitution model was HKY + I + G, aLRT supports are depicted above the nodes. Virus sequence names shown symbols were reference strains of each HRV species with GenBank accession numbers in parentheses. The GenBank accession numbers assigned for the Panamanian samples were JN797533 to JN797594.

Alignment of the sequences in the present study with the HRV reference strains resulted in the three HRV-A, HRV-B, and HRV-C genetic clusters (Figure 1). Higher frequencies of detection were observed for HRV-A (60%, n = 37) and HRV-C (32%, n = 20), over HRV-B species (8%, n = 5). In a recently published study of both children and adults HRV-A was reported as more common than HRV-C in children aged less than 1 year, while HRV-C was the most common species in juvenile patients aged 1 to 19 years [14]. In our study, HRV-A was the most common species in children aged less than 1 year (24 of 44 samples), but also in patients aged 1 to 5 years (13 of 18 samples).

The same region of VP4-VP2 region for each positive sample amplified and sequenced was used to identify the serotypes within each species. To do this, we constructed phylogenetic trees with the dataset of published sequences belonging to all HRV-A, HRV-B serotypes, and also from the recently described HRV-C species variants [9,12].

**Figure 2:**
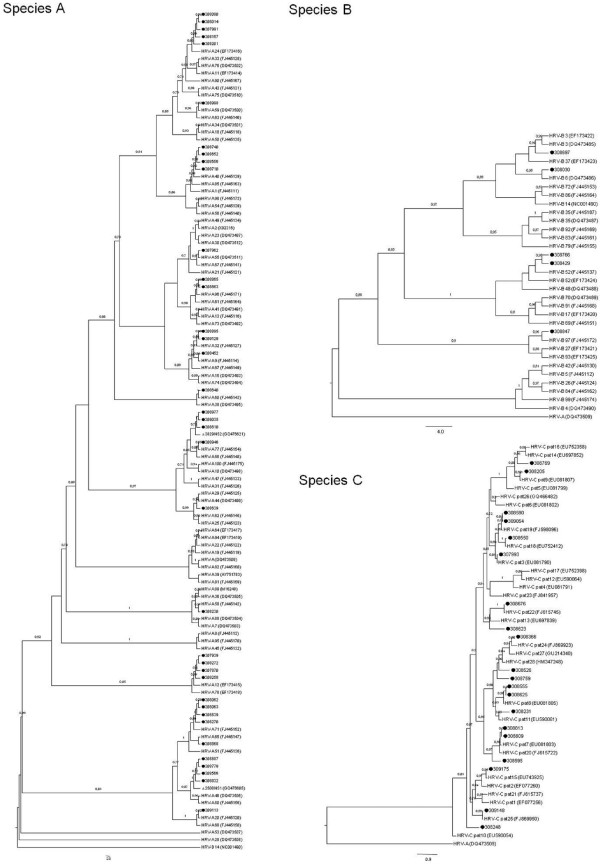
**Phylogeny of the VP4-VP2 sequences amplified of HRV variants detected in the study in comparison of those sequences from serotype reference strains of species A and B and provisionally assigned for species C. **Phylogenies were constructed under maximum likelihood criterion (PhyML software), with the following models of nucleotide substitution: GTR + I + G for species **A **and **B **and HKY + I + G for species **C**. The GenBank accession numbers assigned for the Panamanian samples were JN797533 to JN797594. For species **B **and **C **variants, HRV-A (GenBank accession number DQ473509) was used to root the trees; while sequence HRV–B14 (GenBank accession number NC_001490) was used to root the species **A **tree.

Variants in HRV-C species are not currently assigned to serotypes. However, some attempts to assign types with temporary names based on tree position have been performed [11], and more recently a proposal for the designation of HRV-C provisional types 1–28 has been published, based only on VP4/VP2 sequences [12]. Following the latter classification, the 28 published sequences were used to construct a phylogenetic tree with the 20 HRV-C samples identified in this work. Fourteen sequences were assigned to previously reported types, however, six samples (308526, 308759, 308769, 308595, 308623 and 308248) could not be assigned to any of the 28 provisional types (Table 1, Figure 2-C). These samples exhibited a percentage of p-distance greater than 10% with respect to the 28 HRV-C types. Thus, following the criterion proposed by Simmonds et al., (> 10% of divergence in VP1-VP4 region between different types) the untyped strains could be assigned to new types.

**Table 1:** HRV serotypes detected among study subjects within each species

**Species and serotype**	**No. of samples with the indicated serotype**
***Species A***	
A12	4
A57	1
A24	5
A71	4
A58	1
A9	1
A40	4
A96	2
NS1	4
NS2	3
A60	1
A32	2
A77	1
A59	1
A20	1
Untyped (308639)	1
Untyped (308868)	1
***Species B***	
B3	1
B6	1
B52	2
B97	1
***Species C***	
Pat8	2
Pat11	1
Pat24	1
Pat9	1
Pat7	2
Pat22	1
Pat18	1
Pat3	1
Pat19	2
Pat25	1
Pat15	1
Untyped (308526)	1
Untyped (308759)	1
Untyped (308769)	1
Untyped (308595)	1
Untyped (308623)	1
Untyped (308248)	1
**Total**	**62**

Table 1 and Figure 2-A, show that of 37 HRV-A samples, 35 were assigned to previously reported serotypes, including 7 samples clustering with the recently assigned new HRV-A serotypes, named NS1 and NS2 [11]. Two samples (308639 and 308868) could not be properly assigned to the actually defined serotypes. Sequence 308639 displayed a p-distance greater than 10% with respect to all of the HRV-A serotypes, except for serotypes 29 and 44. Because of this, and following the criterion postulated by Simmonds et al., for HRV-C (> 10% of divergence between types) [12], this strain should not be assigned to a new serotype. Sequence 308868 shows a p-distance greater than 10% with respect to every other serotype. Thus, following the same criterion, this strain could be provisionally assigned to a new serotype.

Five samples identified as HRV-B were clustered into four previously reported HRV-B serotypes (Table 1, Figure 2).

The HRV strains analyzed showed substantial genetic diversity, a fact that is supported by a Shannon entropy analysis performed with the sequences from Figure 1, which displayed values between 0.6 and 1 for the majority of the sites analyzed (data not shown, available on request).

Marked genetic diversity within HRV-A and HRV-C over HRV-B species has been demonstrated in this study, with HRV-B sequences being the most homogeneous species, showing an intra-species nucleotide p-distance of 0.19, followed by rhinovirus A, with 0.20. The most heterogeneous was species C, with a p-distance of 0.25.

The emergence of previously untyped serotypes of HRVs in our study represents a challenge for HRV researches to develop the necessary tools to allow for reliable classifications.

Additional studies involving subject of all ages hospitalized and also ambulatory will be done in Panama, in order to elucidate the relationships between HRV species and to define the epidemiological profile and genetic characteristics of each one.

Panamanian strains were deposited in the GenBank Database under accession numbers [Gen-Bank: JN797533 to JN797594].

## Competing interests

The authors declare that they have no competing interests.

## Authors’ contributions

JA and JP conceived of the study, and participated in its design and coordination. DF, LA, MC, MC, CC and JC carried out the PCR and sequencing studies and drafted the manuscript. AD, JA and DF participated in the phylogenetic analysis and JA wrote the paper. All authors read and approved the final manuscript.

## References

[B1] Andrewes CH The complex epidemiology of respiratory virus infections Science 1964 146 1274 1277 10.1126/science.146.3649.1274 14207453

[B2] Arruda E Pitkaranta A Witek TJ Jr Doyle CA Hayden FG Frequency and natural history of rhinovirus infections in adults during autumn J Clin Microbiol 1997 35 2864 2868 9350748 10.1128/jcm.35.11.2864-2868.1997 PMC230076

[B3] Hayden FG Rhinovirus and the lower respiratory tract Rev Med Virol 2004 14 17 31 10.1002/rmv.406 14716689 PMC7169234

[B4] Mackay IM Human rhinoviruses: the cold wars resume J Clin Virol 2008 42 297 320 10.1016/j.jcv.2008.04.002 18502684 PMC7108405

[B5] Nicholson KG Kent J Hammersley V Cancio E Acute viral infections of upper respiratory tract in elderly people living in the community: comparative, prospective, population based study of disease burden Br Med J 1997 315 1060 1064 10.1136/bmj.315.7115.1060 9366736 PMC2127683

[B6] Boivin G Osterhaus AD Gaudreau A Jackson HC Groen J Ward P Role of picornaviruses in flu-like illnesses of adults enrolled in an oseltamivir treatment study who had no evidence of influenza virus infection J Clin Microbiol 2002 40 330 334 10.1128/JCM.40.2.330-334.2002 11825938 PMC153349

[B7] Bellei N Carraro E Perosa A Watanabe A Arruda E Granato C Acute respiratory infection and influenza-like illness viral etiologies in Brazilian adults J Med Virol 2008 80 1824 1827 10.1002/jmv.21295 18712837 PMC7166366

[B8] Stanway G Brown F Christian P Hovi T Hyypia T King AM Q Knowles NJ Lemon SM Minor PD Pallansch MA Palmenberg AC Skern T Fauquet CM, Mayo MA, Maniloff J, Desselberger U, Ball LA Family Picornaviridae Virus taxonomy 2005 Eighth Report of the International Committee on Taxonomy of Viruses. Elsevier/Academic Press, London, United Kingdom 757 778

[B9] Lau SK Yip CC Tsoi HW Lee RA So LY Lau YL Chan KH Woo PC Yuen KY Clinical features and complete genome characterization of a distinct human rhinovirus (HRV) genetic cluster, probably representing a previously undetected HRV species, HRVC, associated with acute respiratory illness in children J Clin Microbiol 2007 45 3655 3664 10.1128/JCM.01254-07 17804649 PMC2168475

[B10] Huang T Wang W Bessaud M Ren P Sheng J Yan H Zhang J Lin X Wang Y Delpeyroux F Deubel V Evidence of recombination and genetic diversity in human rhinoviruses in children with acute respiratory infection PLoS One 2009 4 e6355 10.1371/journal.pone.0006355 19633719 PMC2712091

[B11] Wisdom A Leitch EC Gaunt E Harvala H Simmonds P Screening respiratory samples for detection of human rhinoviruses (HRVs) and enteroviruses: comprehensive VP4-VP2 typing reveals high incidence and genetic diversity of HRV species C J Clin Microbiol 2009 47 3958 3967 10.1128/JCM.00993-09 19828751 PMC2786677

[B12] Simmonds P McIntyre C Savolainen-Kopra C Tapparel C Mackay IM Hovi T Proposals for the classification of human rhinovirus species C into genotypically assigned types J Gen Virol 2010 91 2409 2419 10.1099/vir.0.023994-0 20610666

[B13] Tapparel C Cordey S Junier T Farinelli L Van Belle S Soccal PM Aubert JD Zdobnov E Kaiser L Rhinovirus genome variation during chronic upper and lower respiratory tract infections PLoS One 2011 6 6 e21163 10.1371/journal.pone.0021163 21713005 PMC3119694

[B14] Fry AM Lu X Olsen SJ Chittaganpitch M Sawatwong P Chantra S Baggett HC Erdman D Human rhinovirus infections in rural Thailand: epidemiological evidence for rhinovirus as both pathogen and bystander PLoS One 2011 6 3 e17780 29 10.1371/journal.pone.0017780 21479259 PMC3066183

[B15] Lu X Holloway B Dare RK Kuypers J Yagi S Williams JV Hall CB Erdman DD Real-time reverse transcription-PCR assay for comprehensive detection of human rhinoviruses J Clin Microbiol 2008 46 533 539 10.1128/JCM.01739-07 18057136 PMC2238069

[B16] Savolainen C Blomqvist S Mulders MN Hovi T Genetic clustering of all 102 human rhinovirus prototype strains: serotype 87 is close to human enterovirus 70 J Gen Virol 2002 83 333 340 11807226 10.1099/0022-1317-83-2-333

